# Biometrics: Going 3D

**DOI:** 10.3390/s22176364

**Published:** 2022-08-24

**Authors:** Gerasimos G. Samatas, George A. Papakostas

**Affiliations:** MLV Research Group, Department of Computer Science, International Hellenic University, 65404 Kavala, Greece

**Keywords:** 3D biometrics, computer vision, 3D reconstruction, identity recognition

## Abstract

Biometrics have been used to identify humans since the 19th century. Over time, these biometrics became 3D. The main reason for this was the growing need for more features in the images to create more reliable identification models. This work is a comprehensive review of 3D biometrics since 2011 and presents the related work, the hardware used and the datasets available. The first taxonomy of 3D biometrics is also presented. The research was conducted using the Scopus database. Three main categories of 3D biometrics were identified. These were face, hand and gait. The corresponding percentages for these categories were 74.07%, 20.37% and 5.56%, respectively. The face is further categorized into facial, ear, iris and skull, while the hand is divided into fingerprint, finger vein and palm. In each category, facial and fingerprint were predominant, and their respective percentages were 80% and 54.55%. The use of the 3D reconstruction algorithms was also determined. These were stereo vision, structure-from-silhouette (SfS), structure-from-motion (SfM), structured light, time-of-flight (ToF), photometric stereo and tomography. Stereo vision and SfS were the most commonly used algorithms with a combined percentage of 51%. The state of the art for each category and the available datasets are also presented. Finally, multimodal biometrics, generalization of 3D reconstruction algorithms and anti-spoofing metrics are the three areas that should attract scientific interest for further research. In addition, the development of devices with 2D/3D capabilities and more publicly available datasets are suggested for further research.

## 1. Introduction

Biometrics are unique body characteristics used to identify people. They were first used at the end of the 19th century with the well-known and globally used fingerprints. According to Jain et al. [1], the most commonly used biometric methods are DNA, ear, facial, hand and finger veins, fingerprint, gait, hand geometry, iris, palmprint, retina, signature and voice. To perform the identification, a device is used to capture the biometric data. Most often, this device captures images, and the quality of the captured images therefore affects the performance of the model. The first identifications were completed manually by experts, but in some cases, the results were controversial due to the human factor. Later, the use of technology for identification has evolved in the form of image processing methods and matching techniques, creating tremendous identification models that take advantage of biometrics. Over the years, these technologies achieved great performance and thus became more popular. Moreover, biometrics for identifying people is nowadays not only used in forensics but also to gain access to certain places or to log in to some smart devices.

As technology has advanced, a biometric security problem has emerged. The technology became very familiar and very vulnerable to malicious acts. This has had a major impact on various security protocols as biometrics have become a part of our daily lives. To counter this, a few approaches have been developed in this area. One of them is to increase the robustness of the selected biometric category. Bear in mind that the traditional methods are referred to 2D, and some scientific approaches have led to the development of 3D biometrics. Of course, this was not just about the fancy addition of the third dimension but mainly about increasing the extracted features and creating more efficient systems. These additional features are the key to the desired performance improvement.

Computer vision has always been linked to biometrics as it provides the necessary tools for identification through 3D image analysis. In addition, the technological advancement of computer vision using state-of-the-art Artificial Intelligence methods to achieve the above benefits has led to the need to apply it to identification systems. As the demand for robust models has increased, the transition from 2D to 3D biometric methods has been a one-way street.

**Figure 1:**
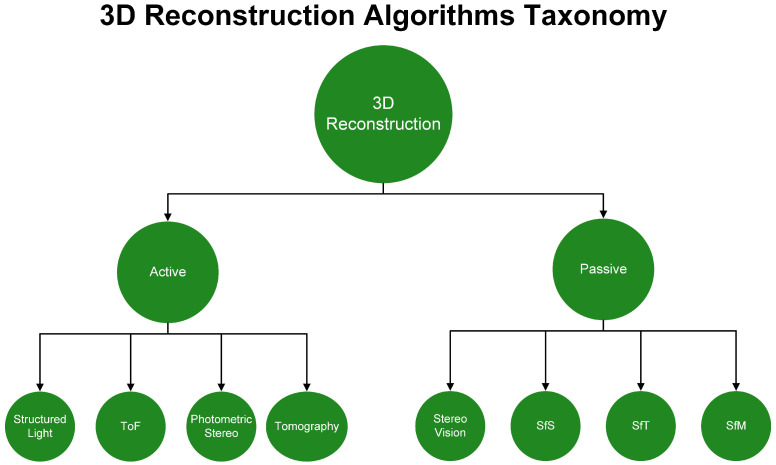
Three-Dimensional (3D) Taxonomy.

The core element for 3D reconstruction is depth information. Various algorithms have been developed to extract the relevant information. The first work published for 3D biometrics in general was from David Zhang and Guangming Lu in 2013 [2]. In their book, they described the image acquisition methods and categorized them into two major categories, the single and multi-view approaches. Another approach is to categorize them into active and passive methods. In active methods, the light source is directed to the desired surface as an essential step for 3D reconstruction and is characterized by low computational cost. Passive methods, on the other hand, are usually very computationally intensive and use ambient light conditions [3]. Furthermore, active methods can be categorized into structured light, time of flight (ToF), photometric stereo and tomography. Passive methods include stereo vision, structure-from-silhouette (SfS), structure-from-texture (SfT) and structure-from-motion (SfM). All of the above methods are presented in the form of a taxonomy in Figure 1. The taxonomy shows that the two main approaches, active and passive, have four methods at once. With further approaches, it is possible to create a third category. This category could be semi-active, semi-passive, or even a combination of passive and active methods. Such multimodal approaches should lead to new 3D reconstruction algorithms.

Structured light produces a beam from a light source onto the surface of the object. The wavelength of the light can be in the visible range, the infrared (IR) or even the near infrared (NIR). The calculations from the reflection of the beam provide depth information. Secondly, the ToF method takes into account the reflection time between the surface and a reference point, while the photometric stereo method uses different lights to create different shades of the object and the model is created by combining these lights. Finally, tomography can be either optical coherence tomography or computed tomography (CT). In both cases, the 3D models are created by multiple scans.

In addition, stereo vision creates depth information by comparing image information of the same spot from two different viewpoints. SfS uses images taken from different angles to form the silhouette of the object. In addition, the SfT is applied when the surface has a homogeneous texture and then uses the different orientation of the images to create the depth details. Finally, the SfM uses a video as input, which is usually consisting of frames that capture an object from different angles.

An important factor for successful reconstruction is the sensor used. The RGB-D camera is commonly used in various 3D reconstruction applications. This particular type can provide color and depth information by combining the three primary colors (red–green–blue) and calculating the relative distance to the sensor accordingly. According to an analysis of RGB-D camera technologies for face recognition, Urlich et al. [4] found that stereoscopy (active and passive), followed by structured light, produced the best results. The importance of these cameras was also emphasised by Zollhöfer et al. [5]. In the research, they presented the different approaches and the great performance of these sensors for all aspects of 3D reconstructions, including biometric elements such as the face, etc.

Furthermore, the above eight different methods are used throughout the literature without any correlation between the categories of 3D biometrics, as each category has been studied separately so far. Although some reviews refer to a group of categories, such as facial and ears, the vast majority refer explicitly to one category. This creates additional barriers to the extraction of cross-biometric information, such as common methods between categories or even similarities in the state of the art. Furthermore, there is no literature review in the field that examines all categories of 3D biometrics at once.

This review examines 3D biometrics through a literature review of scientific work in the field, focusing on 3D reconstruction algorithms and methods. Both are essential for successful identification with 3D images. Furthermore, the contribution of this work is to provide statistical data on 3D biometrics so that a qualitative and quantitative comparison of each biometric category and its 3D reconstruction methods is possible. The result is the first taxonomy of 3D biometrics and a correlation analysis between 3D reconstruction methods and biometric category. The analysis shows that face as a 3D biometric is over-saturated with a variety of approaches in this direction. Moreover, stereo vision and SfS are the most commonly used methods among biometric categories. The analysis also presents various available datasets and the state of the art in each category. Finally, it highlights the challenges that have arisen and need to be addressed in the transition to 3D.

The paper is organized as follows: Section 2 contains the related work on reviews about the 3D biometrics, Section 3 presents the literature search protocol, which is vital prior to gathering firm results through literature searching. Section 4 presents the 3D reconstruction approaches for the three main biometric categories of face, hand and gait. The categories and their subcategories were extracted through the literature search. Section 5 contains the results of the research with the available datasets, state of the art and statistics. Section 6 contains a discussion of the work, indicating the need for further research and suggestions. Finally, Section 7 contains the conclusion of the paper, summarizing all the information provided in the previous sections.

## 2. Related Work

In order to document the related work, a preliminary research was conducted using the trusted platform Scopus [6]. A customized search query was applied. More specifically, the word 3D biometric was selected, and the results were limited to certain types of publications (reviews, survey etc.) The research revealed that the published 3D biometrics reviews consist of two main categories: the face and fingerprints.

### 2.1. Face

The first and most popular category was the face recognition, with the facial and ear being two popular subsections. In some cases, the ear is part of a multimodal approach or a standalone biometric. In 2011, Yuan et al. [7] studied ear recognition. In the article, the author had also included the recognition process of 3D images for the first time. The ear has a unique shape and also shows minor deformations over the years. According to the article, the main reconstruction methods are SfS, SfM and Stereo Vision, with the last method being the most effective. Yaun et al. [7] also conclude that the accuracy and robustness of the system are greatly improved when ear features are used in combination with face features.

The first review for facial biometric was by Islam et al. in 2012 [8], which presented a 3D facial pipeline in four stages. These stages were 3D data acquisition, detection, representation and recognition. The authors provide some details about the data acquisition techniques. For face recognition, there are two main categories: the use of 2D or 3D images. The state of the art for using 2D images was the Support Vector Machine (SVM) with 97.1% on 313 faces [9] and for 3D images was the Point Distribution Model (PDM), which achieved 99.6% on 827 images [10]. For the representation of the face, the balloon image [11] and iso-countours [12] were the most advanced models. When used to reconstruct a face, they achieved 99.6% and 91.4% accuracy in face recognition, respectively. The final step was the recognition. Since the face often changes during emotional expressions, the author presented two main categories: rigid and non-rigid, depending on whether the model is considered rigid or not. Although the percentages for the rigid approach were high (the Iterative Closest Point (ICP) algorithm [13] reached 98.31%), some samples were rejected by the algorithm due to different expressions. On the other hand, rigid approaches had similar performance but increased computational cost. For ear reconstruction, the author proposed three different approaches. The first was to use landmarks and a 3D mask, but this approach depends on manual intervention. For the second approach, the use of 3D template matching was proposed, with an accuracy of 91.5%. This approach had better performance than the previous one but a higher error rate. The last and most efficient method was the Ear Shape Model, proposed by Chen and Bhanu [14] in 2005, which achieved an accuracy of 92.5% with an average processing time of 6.5 s.

The use of ToF methods in 3D face recognition was reviewed by Zhang and Lu in 2013 [2], presenting two main approaches for ToF applications. The first is image capture with incoherent light, and the second is based on optical shutter technology. Both use the reflection of NIR light. They also pointed out the disadvantages of these devices, namely the high error rates due to the generation of low-resolution images by the different devices. Of course, they also believe that hardware will be able to support a higher resolution biometric system in the near future. The main advantage of the Tof is that it can provide real-time results, which are very important for biometrics.

In 2014, Subban and Mankame [15] wrote a review paper focusing on 3D face recognition methods and proposing two different approaches. The first extracts features from the different facial attributes (nose, lips, eyes, etc.), and the second assumes the face as a whole entity. Furthermore, the authors presented the methods that had the best performance based on recognition rate (RR). In particular, a combination of geometric recognition and local hybrid matching [16] achieved 98.4%, which was followed by the method of local shape descriptor with almost the same performance (98.35%) [17]. The remaining methods 3D morphing [18] and multiple nose region [19] were equally efficient with 97% and 96.6%, respectively. In the same year, Alyuz et al. [20] described the phenomenon of difficulty in identifying 3D faces in the presence of occlusions. These occlusions can be accessories such as hats or sunglasses or even a finger in front of the face. However, Alyuz proposed a method consisting of removing occlusions and then restoring the missing part of the face, achieving a high identification accuracy (93.18%).

In the following year, Balaban et al. [21] reviewed deep learning approaches for facial recognition. The author emphasized that as deep learning models evolve, better datasets are needed. In fact, the state-of-the-art Google FaceNet CNN model [22] had an accuracy of 99.63% in the Labeled Faces in the Wild (LFW) dataset [23]. This very high accuracy somehow shows that the scientific community should create datasets with a lot of additional images. Balaban also believes that such a dataset will be revolutionary and compares it to the transition from Caltech 101 to Imagenet datasets. The next year, in 2016, Liu et al. [24] presented the weaknesses of facial recognition systems when the input images are multimodal. These can be IR, 3D, low-resolution or thermal images, which are also known as heterogeneous data. Liu also suggested that future recognition algorithms should be robust to multimodal scenarios in order to be successfully used in live recognition scenarios. The authors also highlight the fact that humans can easily perform face recognition with multimodal images. In order to mimic this behaviour, one approach is to make the different models exposed to long-term learning procedures.

Furthermore, Bagga et al. introduced a review of anti-spoofing methods in face recognition, including 3D approaches [25]. In face spoofing, the “attacker” creates fake evidence to trick a biometric system. In 3D, these proofs are fake masks created from real faces. Four techniques are proposed: motion, texture, vital sign and optical flow-based analysis. Motion-based analysis involves analyzing the movement of different parts of the face, such as the chin or forehead, so that any attempt at forgery can be detected. The best approach is a calculation estimate based on illumination invariance by Klaus Kollreider et al. [26]. The second technique extracts the texture and frequency information using Local Binary Pattern (LBP), which is followed by histogram generation. Finally, an SVM classifier classifies whether the face is real or fake. The third method is vital sign recognition analysis. This can be performed either by user interaction such as following some simple commands (e.g., head movements, etc.) or in a passive way, i.e., by detecting mouth and/or eye movements. Lastly, the optical flow-based analysis, proposed by Wei et al. [27], is primarily used to distinguish a fake 2D image from a 3D face.

In 2017, Mahmood et al. [28] presented the state of the art in face recognition, using 3D images as input. According to their review, five recognition algorithms stood out for their high performance. These are ICP [29], Adaptively Selected Model Based (ASMB) [30], Iterative Closest Normal Point (ICNP) [31], Portrait Image Pairs (PIP) [32] and Perceived Facial Images (PFI) [33] with 97%, 95.04%, 99.6%, 98.75% and 98%, respectively. The authors concluded that despite the high accuracy achieved by applying state-of-the-art algorithms, the models are not yet reliable enough to process data in real-time. Furthermore, they underlined that the performance varies greatly on different datasets, and further research should be conducted in this direction.

Later, in 2019, Albakri and Alghowinem [34] reviewed various anti-spoofing methods. According to their study, pulse estimation, image quality and texture analysis are the most efficient methods. Meanwhile, they proposed a proof-of-concept study in which the fake input is detected by computing the depth data. Using four different types of attacks (flat image, 3D mask, on-screen image and video) on three different devices, iFace 800, iPhone X and Kinect, they managed to detect the fake attack with very high accuracy (92%, 100% and 100%, respectively). The last two reviews were also related to anti-spoofing approaches. Wu et al. [35] focused on applications in China and also presented three trends for protecting against facial forgery. The first was a full 3D reconstruction of the face, whose main drawback is low performance. The next was a multimodal fusion approach consisting of a combination of visible and IR light generated by binocular cameras. Finally, the generative model with a proposed new noise model [36] was the state of the art. Finally, Liu et al. [37] reviewed the results of the Chalearn Face Anti-Spoofing Attack Detection Challenge at CVPR2020. Eleven teams participated using the metric ACER standardized on ISO and achieved great performance with the best percentages of 36.62% and 2.71% for single and multimodal approaches, respectively.

### 2.2. Fingerprint

The 3D fingerprint was first reviewed in 2013 by Zhang and Lu [2]. First, they present the general flowchart of 3D fingerprint reconstruction from images acquired by touchless devices. The first step is to calibrate the camera, which is a very common procedure for reconstruction applications. The next step is to determine the three different correspondences. These are based on the SIFT feature, the ridge map and the minutiae. After these correspondences, the coordinates are created based on the generated matching points. The final step is to produce an estimation of the shape of the human finger. The author concludes that the most difficult process in the whole reconstruction is the establishment of the different correspondences.

In 2014, Labati et al. [38] presented a review on contactless fingerprinting as a biometric. The paper described the advantages of capturing the corresponding images without contact between the finger and the capture device. The author presented three main approaches to reconstruct the fingerprint: a multiview technique, followed by structured light and stereophotometry. In the multiview technique, several cameras are used to obtain the images. More specifically, two, three and five cameras have been proposed. It should be noted here that although a large number of cameras means higher accuracy, it also increases the computational cost. Therefore, the optimal way to design a system is to find a compromise between accuracy and computational cost. Using a structured light provides an estimate of the ridges and valleys of the fingerprint in addition to texture estimation. The light is projected onto the finger several times in the form of a wave to capture the corresponding image. The last method was photometric stereo imaging. Using a single camera and several LED lights as illuminators, the fingerprint was reconstructed using the photometric stereo technique. The above approaches were promising and opened up new scientific fields. The author also emphasized that there is no work yet on the 3D reconstruction of fingerprints that can be used as a biometric.

Later, Jung et al. [39] suggested ultrasonic transducers as a possible way to find depth information. More specifically, the author reviewed the various ultrasonic sensors used in microelectromechanical systems (MEMS) and their applications. One of them was to acquire appropriate depth information of a fingerprint and its use as a biometric. With the improvement of MEMS technology, ultrasonic sensors are improving in terms of accuracy and robustness. Moreover, this type of sensor outperforms the more traditional ones, such as optical or capacitive, achieving better performance. For this reason, Jung underlined that the above sensors have great scientific potential for 3D biometrics. The use of ultrasound devices was further highlighted in 2019 by Lula et al. [40]. The article described the use of ultrasound devices as a suitable method for capturing 3D fingerprints that can be used in biometric systems. The main problem with this approach was the long acquisition time of the images. This was overcome by using additional probes, which are usually arranged cylindrically. It should also be noted that the frequency bandwidth varies between 7.5 and 50 MHz and depends on the probe model and circumference chosen. A high ultrasound frequency offers high skin penetration but low resolution, while a lower frequency has the opposite effect.

Finally, Yu et al. [41] presented a literature review on 3D fingerprints as a biometric using optical coherence tomography (OCT) as an acquisition device. To calculate the depth information, the light beam was directed at the subject and a mirror. The light penetrated the finger and the system correlated the reflection of the finger’s and the mirror’s beam. Through this process, the system calculated the depth information and then reconstructed the 3D representation. The light penetration also provided the inner fingerprint, which is unaffected by environmental influences. Sweat pores and glands are also visible through this approach. These additional elements provide more features, and the biometric system has become more robust as a result. Despite the fact that OCT has the above advantages, there are also some disadvantages. These include latency, cost, mounting limitations and low resolution

Related work shows that only two 3D biometric categories have been reviewed: the face and the fingerprint. This is probably because these biometrics are very common as 2D biometrics, especially facial. This led to the fact that the other biometric categories such as iris or finger vein were not reviewed. In order to present their state of the art, additional reviews about them should be conducted. In this paper, the taxonomy of 3D biometrics is first created, and then, its applications are examined. As a result, the state of the art and common 3D reconstruction methods between the categories can be presented.

## 3. Literature Search

In order to obtain additional information on further advances in 3D biometrics, a literature analysis was conducted. This section contains that analysis with two subsections: the search protocol and the initial statistical results. It should be mentioned here that the search was conducted according to strictly defined criteria so that everyone can reproduce the methodology used and obtain the same results.

### 3.1. Search Protocol

In order to obtain usable results, a search query was carefully designed and also applied to Scopus. The following query was applied simultaneously to the abstract, title and keywords of the publication:
3D* W/2 reconstruction*ANDbiometric*ANDPublication Year > 2011

The term “W/2” was inserted to ensure that the “3D” and the “reconstruction” were close in the paragraph (two words). The “*” ensures the singular or plural form of the words “biometric” and “reconstruction”. The total number of articles researched was 96. The first problem that arose early on was correspondence with biometrics in general. More specifically, many medical publications did not refer to biometrics despite references to 3D reconstruction algorithms. This was due to the misleading double meaning of the word “biometric”. To counteract this, a quality criterion was applied that excluded the above-mentioned publications. As a result, 31 papers were removed. In addition, 11 articles could not be found or the language was not English, and one article was a duplicate entry. The total number of papers that were thoroughly reviewed was finally 53.

### 3.2. Statistical Results

**Figure 2:**
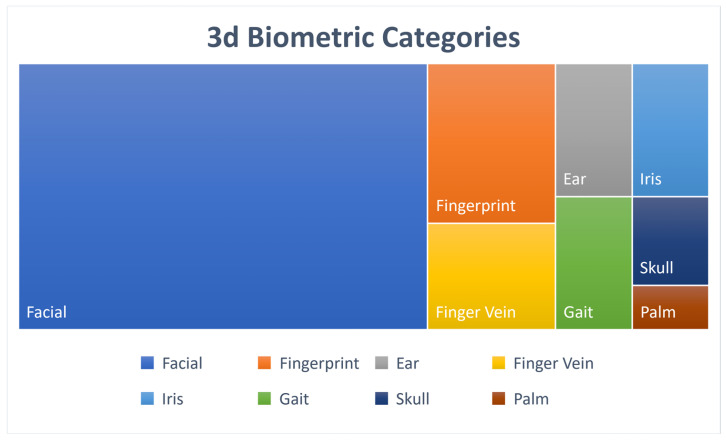
3D Biometric Treemap.

**Figure 3:**
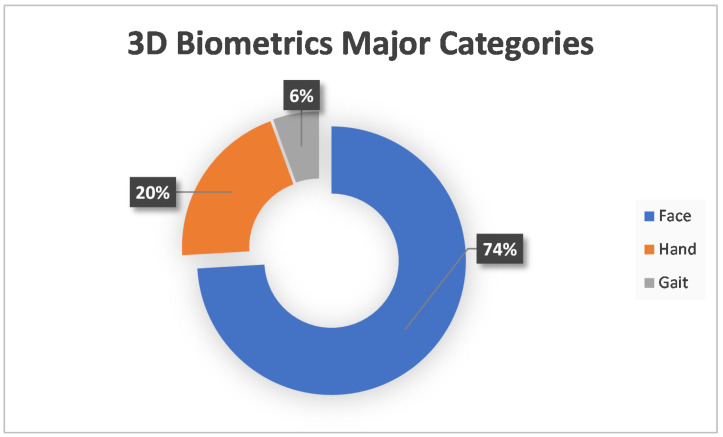
Three-Dimensional (3D) Biometrics Major Categories.

**Figure 4:**
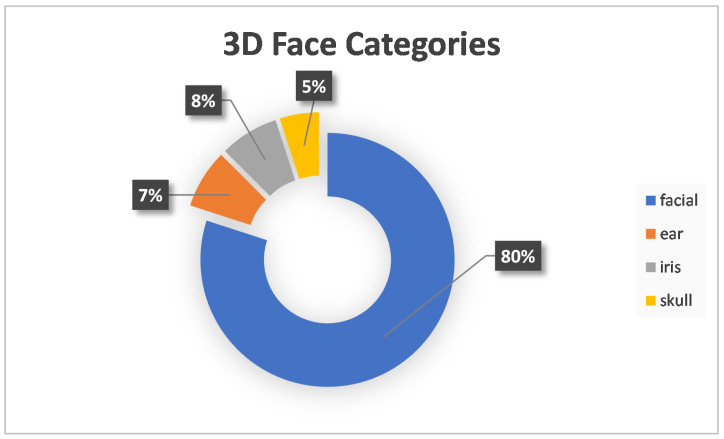
Three-Dimensional (3D) Face Categories.

**Figure 5:**
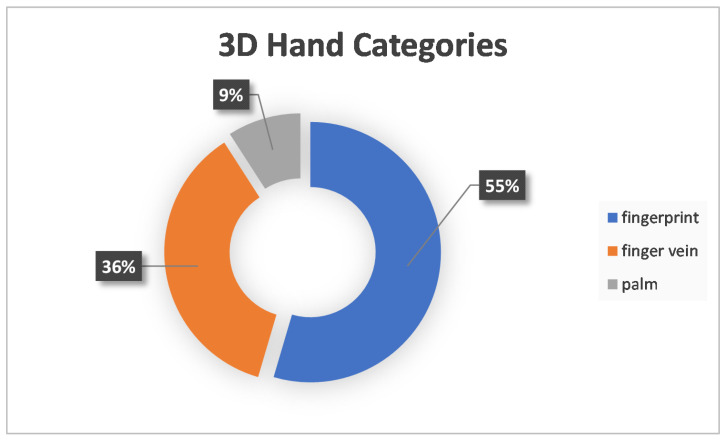
Three-Dimensional (3D) Hand Categories.

At first, the most important result extracted was the 3D biometrics categories. Three main categories were extracted: face, hand and gait. The first two had further subcategories. More specifically, for the face, there was the facial, ear, iris and skull, and for the hand, the categories were fingerprints, finger veins and palm. Considering that one paper experimented with two different biometrics, the total number of approaches was 54. The following Figure 2 shows a tree diagram with the number of applications of all the categories of 3D biometrics described above, while Table 1 shows the main category, percentage and corresponding citations for each biometric feature. In addition, Figure 3 shows a pie chart with the number of applications of the three main categories. Two other pie charts (Figure 4 and Figure 5) also contain the number of applications for the face and hand category, respectively.

## 4. Three-Dimensional (3D) Reconstruction

**Figure 6:**
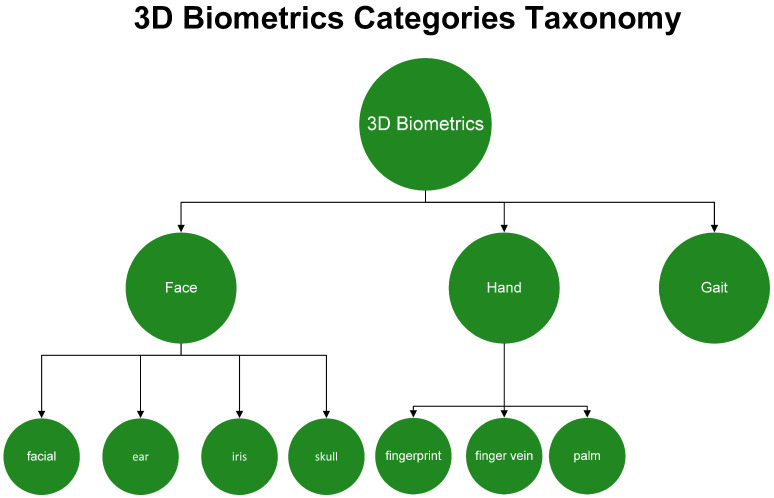
Three-Dimensional (3D) Biometrics Categories.

**Figure 7:**
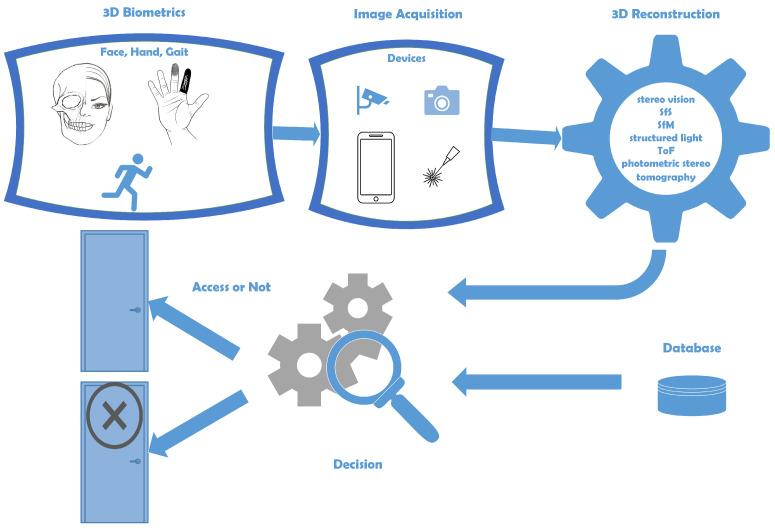
Three-Dimensional (3D) Biometrics General Flowchart.

This section presents the applications obtained from the literature analysis, focusing on the 3D reconstruction approaches for each 3D biometric category. Since the face subcategory contains the most applications and is saturated and over-described as 3D biometric, it is not presented in as much detail as the other categories (relative to the number of applications) in this paper. As mentioned earlier, there are three main categories of 3D biometrics: the face, the hand and the gait. Figure 6 shows the taxonomy of these categories with their subcategories. The taxonomy, which is the first attempt at 3D biometrics, shows that not all biometrics are 3D. Some other biometrics such as voice, heartbeat or DNA should be further explored to determine if they benefit from becoming 3D. A general flowchart of 3D biometrics is proposed in Figure 7. The selection of the appropriate biometric is followed by image acquisition. This is followed by 3D reconstruction using one of the available methods. The system compares the 3D model with the data in the database and decides whether the identification of the person is valid or not.

### 4.1. Face

Face is the first and most popular category. Its subcategories are the facial, the ear, the iris and the skull.

#### 4.1.1. Facial

Facial recognition is the predominant method for 3D biometric applications according to the above research. To be used as a biometric, a 3D model must first be created, which is followed by feature extraction. This can be completed using computer vision techniques. The way the images are captured determines the reconstruction method. Of the total 32 papers, more than half (18) used an active method. This corresponds to 56.25%. The remaining 14 are divided as follows: 10 of them (31.25%) used passive methods, three had used a public dataset (9.38%), and one did not refer to the method used at all. Analyzing the passive methods further, the results show that three subcategories are related to facial recognition: stereo vision, SfS and SfM. The percentages were almost equal as seven applications used SfS, six used stereo vision and five used SfM. The majority of active approaches (9) used reflection from a light source, and only one used transmissive computerized tomography. In addition, the reflective methods can be further divided into two subcategories: structured light and time-of-flight. These percentages were also similar, with five using structured light and the rest (four) using time-of-flight.

Another feature of 3D reconstruction is the way in which depth information is represented. The vast majority of applications used a 3D Morphable Model (3DMM) or point clouds. The total percentage was 78.1% for both, which was split into 40.6% and 37.5% for the point cloud and 3DMM, respectively. The remaining 25% were distributed as follows. Four used third-party software, two used unique approaches, and one was without any information. The approach using the 3DMM used a pre-created 3D model, specifically a face, which is very common in the scientific community. A very robust and effective model is the 3DMM proposed by Chu et al. [92]. These models were created by averaging values of different facial features. In most cases, these values were manually inserted, while the final 3D model is created by fine-tuning the above values. The third party software was thatsMyFace.com [42], Amira version 5.2.2, Visage Imaging, San Diego, CA, USA [43] and MeshLAb [59].

In addition, Dou et al. [52] have proposed Deep Recurrent 3D FAce Reconstruction (DRFAR). The network combines a set of Deep Convolutional Neural Network (DCNN) and Recurrent Neural Network (RNN). Prior to the training, the authors transferred the weights of the pre-trained VGG-Face [93]. This neural network architecture was able to achieve good results after experiments in different databases with three to six face images. In addition, Kneis et al. [67] in 2020 presented an alternative method to synthesize a 3D image from four different images of the same subject with different illumination and achieved excellent performance.

Finally, Crispim et al. [59] used a state-of-the-art approach to verify kinship between two faces. They used the SfM approach. Video input was captured with a smartphone camera in an uncontrolled environment. Then, the images were aligned and cropped to 64 × 64 pixels. Feature detection and extraction were followed by feature matching and geometric verification, and finally, projection of the model into a point cloud. The final part was classification, which relied on a two-layer CNN with the ReLU activation function. The highest score achieved with the comparison between the daughter and mother with the accuracy was 95%. To make the dataset more robust, the authors synthesized non-relative faces through generative adversarial networks by combining two different faces. As mentioned in the previous sections, facial is overused. Due to the high number of applications, a lot of effort was put into it using new approaches. CNNs are already used for classification purposes. This makes it necessary to modify the quality criteria of the proposed datasets, as this type of model is usually designed to perform by using a large number of images.

#### 4.1.2. Ear

The next subcategory is the ear. This category shares characteristics with facial recognition. Although ear recognition is strong enough to be used as a stand-alone biometric, some researchers use ears as a complement to facial recognition [94]. One such approach is by Raghavendra et al. [45], who used facial recognition along with features extracted from the ear. Actually, the authors used the SfM approach prior to the entire face reconstruction. Then, the ear is detected from the reconstructed model and used for identification. The authors also proposed an ear detector using OpenCV 2.4. For training the model, they used 2000 positive and 300 negative samples from existing databases. The results show that the identification rate with the facial as a standalone biometric was 82.35%, which decreases slightly to 80% when fused with the 3D ear. The 3D ear as a stand-alone biometric had a rate of 62.5%, which increased to 80% after fusion.

Furthermore, Siu-Yeung Cho proposed a recognition approach using 3D ear models under different illumination parameters in his paper [82]. More specifically, by applying a generalized neural reflectance (GNR) model, they managed to synthesize ear images. Twenty photographs of each ear were taken from 85 different individuals, and after applying the GNR, 40 additional images were created from each individual image. In this way, a 3D ear database was created. The algorithm chosen was SfS, as the models were created from a single image (synthetic or real). A total of 12 experiments were conducted, and the performance proves that Cho’s GNR model performs significantly better when GNR is applied. Finally, the author suggested the use of principal component analysis (PCA) [95] and Fisher’s discriminant analysis (FDA) [96,97] for classification.

The state of the art is considered to be the approach of Chen Li et al. [98], in which they proposed a novel method for 3D ear reconstruction. First, a 3D Ear Morphable Model (3DEMM) was developed based on the general morphable model [99] containing 180 different ear images. Then, 179 of them were used for training and the last one was used for fitting. In addition, they proposed a novel method for feature correspondence, the Triangle Mesh Hierarchical Growth, which is based on physical features common to each ear. The constructed ear had 10,000 points, which is a higher point density than the SfM and SfS algorithms. The point density as mentioned above can be compared to the use of a laser scanner. It is quite old work, and the lack of modern approaches means that 3D ear biometrics is likely to become extinct in the near future. To avoid this, a new application with new methods and data sets should be carried out. This will certainly bring the ear again in the field.

#### 4.1.3. Iris

The next subcategory is the iris. It owes its biometric properties to the unique surface made of two muscular fibers [100]. In general, there is a lot of scientific research in the field of the iris as a biometric but in two dimensions. Bastias et al. [84] claim that their work is the first approach to create a 3D iris model. In their work, they propose the use of near-infrared (NIR) images taken with a Raspberry Pi v2.1 camera. In addition, infrared light was generated from four NIR LED. To keep the pupil relatively small, two white LED were used simultaneously to create a bright environment around the pupil. This is important because prior to the 3D reconstruction, the pupil is removed, and the depth information is extracted from the rest of the eye. The Raspberry Pi 3 Model B single-board computer was used to control the camera and store the images temporally. All the above components were mounted on a VR glass set. The camera was also on a proposed mount that allowed it to move along an arc of 40${}^{\circ }$. The system was designed to capture a total of 17 iris images, with an angular difference of 2.5${}^{\circ }$ between the two images. In addition, the authors used the Python photogrammetry toolbox [101] to create the 3D model. The toolbox is open-source and creates the model using various images from different angles. It is also noted that it was difficult to evaluate the results and methods of the work due to the lack of reports on metrics and results.

In contrast to the previous approach, Benalcazta et al. [85,86] chose visible light as the light source. They have developed a novel system that illuminates the iris with lateral and frontal visible light (LFVL). The light is produced by six LEDs in the front and two more on the side of each eye. This different angle of the light source creates the shadows and depth information of the iris. The dataset was created from 120 different subjects by recording each eye for three seconds. The total number of images was 26,520. In addition, the authors synthesized an iris dataset consisting of 100 virtual models. To do this, they used Blender, which is an open-source application for creating various 3D shapes. They also augmented the models by generating 720 images per virtual iris, bringing the total number of synthetic images to 72,000. To capture the depth information, they trained a neural network to predict the depth information based on the shadows. More specifically, they proposed irisDepth, which is a combination of the T^2^Net [102] and DenseDepth [103] networks.

The above model has performed excellently compared to other CNN or SfM approaches and is considered state of the art. In fact, the reconstruction accuracy was as high as the reconstruction using OCT. More specifically, when used the irisDepth, they managed to achieve an accuracy of 99.78%. Despite the fact that 3D iris can achieve high accuracy, there are not many applications in this field. This is probably due to the difficult nature of image acquisition. It is not convenient enough to have someone look through a capture device for several seconds to gain access to a location. In the near future, new devices should be developed that allow for easier and more user-friendly image capture so that a 3D iris can be used in real-world problems for recognition.

#### 4.1.4. Skull

The skull, in a biometric sense, refers to cranio facial reconstruction. It has a slightly different scope from the previous one, because it usually involves cadavers, which are difficult to recognize. To aid recognition, a reconstruction of the face is done based on the skull. These algorithms are known as CFR (cranio facial reconstruction), and forensic science is the science that deals with them. A crucial point for successful reconstruction is the facial features introduced by Whitaker and Linton [104]. Their application to cranial reconstruction is used to reconstruct the face of the deceased by combining skull and tissue reconstruction to identify it. Data acquisition also presents some challenges. Early applications used 3D laser scanners, but their performance was limited [105]. Nowadays, therefore, computed tomography (CT) is used. This method gives tremendous results, and the disadvantages are limited to the increased radiation. Therefore, it is preferably applied to non-living organisms [106]. In addition, Vezzetti et al. [107] report that the above landmarks were always set manually.

First, Lorkiewicz-Muszyńska et al. [90] introduced a procedure to identify an unknown deceased person. One way of post-mortem identification is to examine DNA for a match or possible relationship. Another is to examine the dental status of the person. In the case of the paper, neither of the above methods could provide reliable results. More specifically, the DNA examination did not reveal a match with a possible brother of the person, and there were no dental records, either. An identification using a reconstructed skull was chosen to match the person with a photo of a missing person. The SOMATOM Sensation 64 from Siemens was chosen. The In Space software was used for the reconstruction, and the evaluation was done manually by applying the image of the missing person as a surface layer on the 3D reconstructed skull. Finally, the deceased was matched with a missing person photo through the procedure described above. Furthermore, it later turned out that the possible brother had a different father, which is why the DNA examination could not link the deceased to his brother.

The state of the art is indeed skull reconstruction from living humans [43]. Experiments were performed on three volunteers who had not had facial plastic surgery, dental transplantation or general facial deformity. A cone beam computed tomography (CBCT) scanner was used to take several images of the volunteers’ skulls. The model was the Alphard Vega from Asahi Roentgen Co., Kyoto, Japan. Reconstruction was performed using third-party software, FreeForm Modelling Plus^TM^, based on a pre-model database consisting of facial muscles and parotid glands. Finally, model evaluation was performed using deviation maps generated using Geomagic Qualify (Geomagic™ Qualify Version 10; Geomagic, Morrisville, NC) and Rapidform (Rapidform, Seoul, Korea) software. In terms of performance of the proposed method, it achieved the best score on subject B with an average error of 0.31 mm. The other two had 0.46 mm and 0.49 mm for the A and C subjects, accordingly.

The 3D skull biometric is not a very common recognition method. Its uniqueness creates a lot of obstacles. It is used for specific purposes, mostly on cadavers and with an auxiliary function. There is a possibility of creating a framework for skull recognition as a complementary method in forensics in the near future. However, this is not possible today, as more experiments should be conducted to create models specifically for skull reconstruction. Moreover, there is no available dataset, and therefore, future research should be accompanied by the creation of a dataset.

### 4.2. Hand

The second category is the hand, which contains the fingerprint, finger vein and palm.

#### 4.2.1. Fingerprint

Second in order is the most commonly used biometric method in general, the fingerprint. As fingerprints became a biometric identification method used on a daily basis, the need arose to increase the features. This was achieved by replicating the shape of the finger and using the fingerprint as a surface imprint. The 3D fingerprinting methods are also referred to as non-contact methods in the literature [108]. This is very important, because the 2D version of the fingerprint was correlated with the mandatory contact between the finger and the capture device. It is also noted here that much of the problem with 2D fingerprint recognition is related to contact with the device, as there is often dirt, moisture, etc. on the surface of a finger, making identification difficult. A total of six works were found during the research. To obtain the appropriate depth information, four of them used the stereo vision method and the other two used photometric stereo methods and structured light.

Chaterjee et al. [76] proposed a method to reconstruct the fingerprint using a structured light technique. An LED as a light source is projected onto the finger, while a CCD camera records the reflection. More specifically, the projection unit generates sinusoidal fringe patterns, and the frequency selective (FTM) algorithm [109] was selected to obtain the depth information. In order to create a more reliable system, the authors also incorporated biospeckle analysis as an anti-spoofing method. This was achieved by implementing the visual speckle-based MSF algorithm [110] using a laser as the light source. For the experiments, the laser and the LED were mounted in the same device so that both processes (reconstruction and anti-spoofing) could be performed simultaneously. The authors also found that 3D reconstruction outperformed other techniques, and anti-spoofing via blood flow was successful. Consequently, their proposal was simple, fast, cost-effective, and ready to be used as a commercial product.

Another approach to 3D fingerprinting came from Kumar and Kwong [72]. Their approach focused on creating a cost-effective and accurate system. The need to reduce cost stems from the fact that some approaches use multiple or extremely accurate cameras. To overcome this hurdle, the authors used the photometric stereo technique and a digital camera. The finger was placed 10 cm from the camera, and seven LEDs were symmetrically placed. After acquisition, the images were resized from 2592 × 1944 to 500 × 350 pixels. A total of 10,080 2D images of 240 subjects were captured. Seven 2D images were used for each 3D image. In addition, six fingers were reconstructed from each subject. The results show that the 3D approach outperforms 2D finger recognition. The authors also noted that a multimodal approach, combining 2D and 3D features, could provide even better results.

Liu et al. [74] used three JAI CA-A50 cameras along with four blue LEDs. The three cameras were placed below the finger: one in the middle and the other two on each side. The finger was placed on a fixed base in order to be stable throughout the image acquisition process. The reconstruction process was based on stereo vision. In addition, only the two side cameras were used for reconstruction. The central camera is only used for reference and the texture of the fingerprint. Furthermore, the appropriate fingerprint features are based on SIFT [111], ridge maps, and minutiae. SIFT is a popular approach that is robust to deformation variations and low-quality images. The ridge map is an image where the ridge has a value of 1 and the background has a value of 0. Three steps are required to create the ridge map. These are preprocessing, enhancement and post-processing. Preprocessing involves extracting the region of interest (ROI) and normalizing the image. In addition, the author used Gabor filtering, orientation estimation, and ridge frequency estimation to enhance the images. Finally, binarization and thinning of the image are applied as post-process procedures before the ridge map is created. Minutiae, the most common feature of fingerprints, were extracted from the ridge map.

The final result of all the above methods was finalized by the random sample consensus (RANSAC) algorithm [112]. In addition, the author proposed a precomputed 3D finger model. In the absence of ready-made 3D finger shapes, they designed a new model using the active structured light method to create it. Four hundred and forty images of 220 different fingers were collected. According to Liu, the best performance was achieved when all of the above feature extraction methods were used, which also highlights the importance of making the fingerprint 3D.

Labati et al. [73] proposed a 3D reconstruction approach that is also based on the stereo vision technique. Two charge-coupled device (CCD ) cameras were placed underneath the finger. An LED array emitting green light was placed between the finger and the camera. The system also had a photocell that triggers the light to reduce the blurring effect. The system was designed to be unaffected by the rotation of the finger. In addition, the prepossessing procedure also included enhancement and 2D mapping. The device was used in 30 volunteers aged 10 to 64 years. The authors created five different databases. Each database had its own unique characteristics and purpose. In addition, after calculating the depth value using the triangulation method, the 3D point cloud was created. The final step of the process was to apply the fingerprint as a texture to the 3D shape. Finally, Labati used the Neurotechnology Verifinger software and obtained similar results compared to touch-based approaches. As a result, the author stated that more effort for non-contact systems will soon give better results.

Furthermore, two other approaches [75,77] also used stereo vision. The first is by Xu and Hu, who proposed a 3D reconstruction of minutiae using a differential evolution algorithm [113,114]. The authors believe that SIFT is not powerful enough for 3D fingerprints. They also used the Verifinger software to extract the minutiae features. The experiment was conducted with 150 volunteers (10 fingers per volunteer). Three cameras were also used to take pictures from the left, front and right. Although Xu proved from images that their approach found more corresponding points compared to SIFT, the reconstructed 3D minutiae are far from reliable, and further research should be conducted.

The second approach of Yin et al. [77] is the state of the art. In their work, they proposed a very robust system that also had better accuracy and shorter processing times than other modern approaches. The reconstruction was based on the ridge–valley contrast. First, the depth information was extracted using stereo vision. Then, the fingertip regions were extracted using a pre-trained CNN [115], and the images were subsequently rectified. The SIFT method [116] was applied to the images, which was followed by the creation of the ridge maps. Using the disparity map, the 3D depth information was calculated to reconstruct the fingerprint. The authors also proposed feature extraction from the minutiae and 3D topology polymer (TTP), which is a novel method. For their experiment, they used 60 samples from 24 different fingers. To evaluate the performance of the model, they conducted several experiments with different databases and methods, and the model of Yin et al. [77] still achieved better results. More specifically, the average accuracy on both databases, DB1 and DB2, was 98.05%. This result was the best compared to other approaches. In summary, 3D fingerprinting appears to be prosperous, with databases already available to train new models. Future researchers will take advantage of the already existing datasets and implement modern methods, and algorithms and will surely achieve great results. A big advantage of fingerprints is that they are already known to users. Once the hardware improves for better processing time and the accuracy is better than 2D, 3D fingerprints will prevail over 2D.

#### 4.2.2. Finger Vein

The finger vein is an alternative biometric method and has some unique features. First of all, it is robust to spoofing attacks due to the obligatory existence of an IR light source. The difference between the hemoglobin and the skin’s absorption of the IR light creates the shape of the veins, and their unique shape provides the information for identification [117]. Two-dimensional (2D) finger vein identification provides promising results, such as the approach of Zhang and Wang [118], which achieved a recognition rate of over 99% for the USM database [119]. Despite the very high accuracy of 2D recognition, some researchers have already moved to 3D finger vein recognition, taking advantage of the additional features of the third dimension. In contrast to the numerous research papers for 2D finger veins, there is not much related work for 3D finger veins.

First, Ma et al. [79] used the stereo vision method for 3D reconstruction. Two simple CCD cameras, sensitive to IR light, were mounted in opposite directions from an array of NIR LEDs. Then, the captured images were processed using the contrast-limited adaptive histogram equalization (CLAHE) [120], a 5 × 5 denoising median filter and a linear gray transformation. Sequentially, the finger veins and contours were extracted by applying masks and an adaptive threshold algorithm. The stereo vision algorithm generated the appropriate 3D cloud points, and the ICP algorithm was used for matching. An error threshold generated by the least mean square error was made adjustable for different sensitivity requirements. The computational cost depended on the number of iterations before identification. As the authors pointed out, more iterations meant more computational time and more effort could be put into this.

Veldhuis et al. [80] also used the stereo vision approach for reconstruction. In their preliminary study on 3D finger veins, they also used three cameras. In contrast to the approach of Kang et al., the cameras were placed under the finger. Several NIR LED strips used for IR illumination were positioned in the opposite direction to the cameras. A Raspberry Pi single-board computer was also installed to control the different illumination options. Their research focused mainly on 2D finger veins, which is not covered in this article.

Kang et al. [78], reconstructed the finger vein along with the shape of the finger. First, they proposed a prototype acquisition device. Three simple USB cameras were mounted symmetrically to form an equilateral triangle. There were also three NIR LEDs (850 nm wavelength) between the cameras. The function of these cameras was controlled by a Light Control Unit (LCU). The author proposed a 3D reconstruction of the finger shape and used the vein information as texture. The reconstruction was based on a novel 3D finger model (3DFM) developed by the research team. Furthermore, the features were extracted from the vein texture and finger geometry by a specially developed CNN, and after a fusion process, the model generated a matching score. Despite the promising accuracy of the model, the authors emphasized that future research should be conducted to reduce the high processing time and create a more robust model.

Their research continued in 2022; they proposed a state-of-the-art approach to the same problem [81]. The hardware was the same: three cameras next to NIR LEDs to create a finger and apply the veins as texture. The main difference was the reconstruction algorithm, as SfS was used instead of a stereo vision approach. This approach proved to be more efficient and requires less computation time, so the model is able to solve problems in real-time. They also proposed a new detection method called 3DFVSNet. The experiments were conducted with 905 different fingers. Fourteen images were taken of each finger, combining three different rotations. The total number of different images was thus 12,670. The results show that the 3DFVSNet performs better compared to other known approaches, achieving higher accuracy and lower computational costs. The metrics ER and HR were used by the authors. On both databases, the model manages to achieve on both metrics the best result: 2.61% (ER) and 5.07% (HR) for the SCUT-3DFV-V1 and 2.81% (ER) and 4.49% (HR) for the LFMB-3DPVFV. Finger veins have just started to become 3D. The great advantage of this category will become apparent as more research is conducted. Machine learning is already being used. Three-dimensional (3D) finger veins will be evolved when a variety of publicly available datasets and new devices are created that are accurate and fast enough to be used for real-world problems.

#### 4.2.3. Palm

The last biometric hand category is the palm. Svoboda et al. [91] used a not so common 2D biometric, the palm. They also explored the identification performance of the palm (as a biometric) after it became 3D. The structured light technique was used to capture the biometric information from the surface of three palms. The light source was four 10 mW lasers with a wavelength of 532 nm, each of which could produce a single line beam. There were also three green lenses for illumination. Images were captured by two webcams (Microsoft Lifecam HD 3000) mounted on each side. All the above components were controlled by a microcontroller (MC9S08JS16CWJ). In addition, the system was designed to use only a single image from each laser reflection. The depth information was also generated by the triangulation principle, and the root mean square error (RMSE) was used to evaluate the reconstruction, achieving a merged score of 0.0018 on models 2 and 3. The authors emphasize that the best performance was obtained when the information from all four lasers was combined. Finally, they are reluctant to conduct further experiments in this direction to create a more stable model with better accuracy—this further research may or may not reveal the importance of this biometric, because it mainly depends on the convenience of the hardware and the performance.

### 4.3. Gait

Although gait is often used to describe a person’s condition (psychological or physical), it is also used as a unique biometric [121]. As a category, it has no subcategory because it is unique.

Fernandez et al. [87] proposed an approach to 3D gait identification. For their experiments, they used two databases, each with six and 16 cameras. The silhouettes were obtained using the Horprasert algorithm [122], and then, the 3D model was reconstructed using the SfS algorithm. Furthermore, the models were further processed to reduce the features by applying PCA and linear discriminant analysis (LDA). For classification, the authors used SVM. The method gave promising results because a majority-based voting system was applied to achieve perfect accuracy, so further research should be conducted.

The same team [88] proposed a new method a year later to solve the same problem. The main difference between the two approaches was the type of 3D reconstruction. Although the algorithms were the same (Horprasert and SfS), the model was represented as a stack of voxels simulating the person’s gait. The size of the voxels was 0.27 × 10^−4^ m^3^ and was considered by the authors to be sufficient for 3D gait reconstruction. Three gait morphological descriptors were also proposed: cover by rectangles (CR), cover by rectangles projection (CRP) and cover by cubes (CC). The combination of the aforementioned innovations provided more accuracy and robustness for all metrics in both databases.

Finally, Imote et al. [89] presented a state-of-the-art method for 3D gait reconstruction based on the height–constraint assumption, which assumes that the height of body points changes slightly during walking/running. For the experiments, 14 cameras were used with various resolutions (from 960 × 540 to 350 × 240); two of them were controlled by a Raspberry Pi single board computer, while the remaining 12 were controlled by three CCTV recorders. The model had great performance compared to various datasets. The model achieved a better identification rate of almost 95% on the KY 4D database, which was better than other approaches. Moreover, the authors noted the relationship between the number of height points (Np) and the normalized square errors. The greater the number of Np, the fewer the number of errors, with the optimum number of Np being 240. Despite the great performance of the model, the authors mentioned that future 3D gait reconstruction should be conducted on three axes. The first is to make a greater comparison with various approaches, the second is to expand the applicability of the method, making the process more automated, and the third is to make the model more capable of supporting new human activities. Three-dimensional (3D) gait reconstruction will soon be more popular. The great advantage is the simple acquisition method. A few frames are enough to proceed to recognition. The transition to 3D is probably essential because the models should be very robust and benefit from the additional features of the third dimension.

## 5. Results

After reviewing the above papers, a lot of useful information was obtained. The first statistics extracted after the initial literature review were the 3D biometric categories. The most predominant category is facial, which includes 59.28% of the applications. The second category, fingerprint, has a share of 11% and a finger vein of 7.41%. Ear, iris and gait have 5.56%, while skull and palm account for 3.7% and 1.85%, respectively. There are some additional data to the three main categories and Figure 3, Figure 4 and Figure 5. More specifically, the percentages of the three main categories are as follows: face →74.07%, hand →20.37% and gait →5.56%. In the face category, the percentages are as follows: facial →80%, ear →7.50%, iris →7.50% and skull →5%. Correspondingly, for the hand category: fingerprint →54.55%, finger vein →36.36% and palm →9.09%.

Moreover, all datasets used for the above 3D biometrics applications were compiled in Table 2. It consists of 26 different databases grouped according to their biometric category, also indicating the year of publication, the number of images included, and the corresponding citation. It should be noted that the number of images for gait biometric and the Youtube Faces database refers to videos and not images. Table 2 shows that there is an abundance of facial datasets, with the most recent being from 2019, so it is fairly recent. Ear datasets appear to be older. The most recent is from 2012, showing that 3D ears are becoming less common. Although the gait is not very common, there are already five databases. The rather simple way to create such a database has probably led to this high number of databases (related to the number of applications). For the others finger vein, iris and fingerprint, there is one dataset each, but the 3D iris dataset is quite old (2007), and new databases should be created for further development.

In addition, after combining the results of the above literature review in each category, according to the impact on each field alongside citation score and year of publication, a state-of-the-art table is presented as Table 3 grouped by biometric category. It contains also information on the year of publication, biometric category, and correspondence reference.

**Figure 8:**
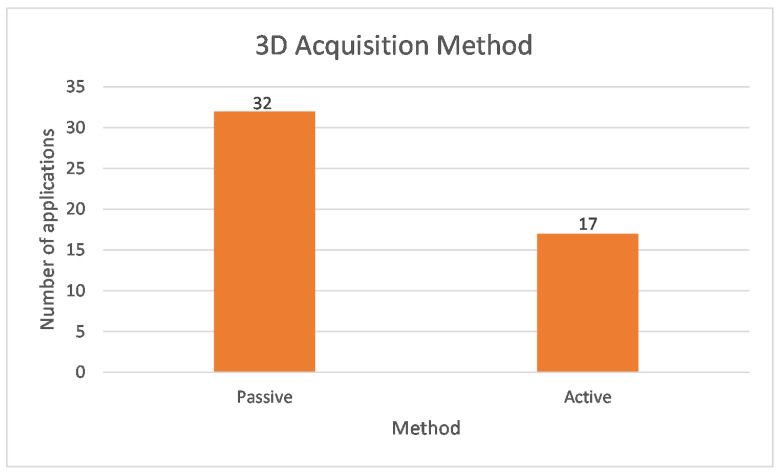
Three-Dimensional (3D) Acquisition Method.

**Figure 9:**
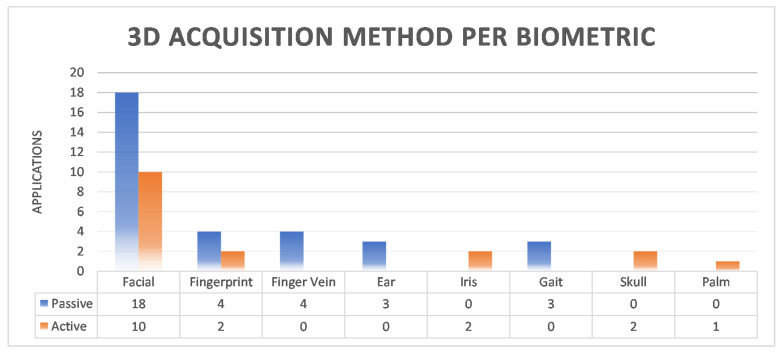
Three-Dimensional (3D) Acquisition Method per Biometric.

Additional information on 3D capture methods was also extracted. Forty-nine applications referred to the selected method for 3D reconstruction. In summary, the results show that 65.3% used the passive method and the remaining 34.7% used the active method, as shown in Figure 8. Further analysis of the percentages per biometric feature is shown in Figure 9. Accordingly, for the facial and fingerprint biometrics, both methods were used, with a slight preference for the passive method. In contrast, only passive methods were used for finger vein, ear, and gait, and active methods were used for iris, skull, and palm.

**Figure 10:**
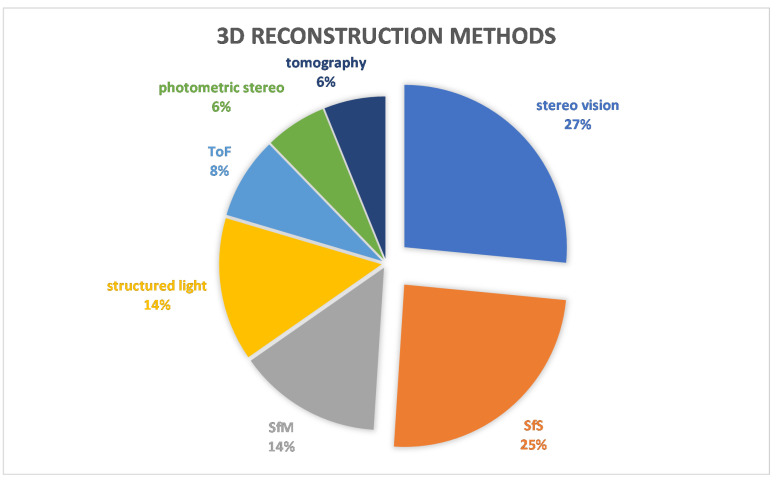
Three-Dimensional (3D) Reconstruction Methods.

**Figure 11:**
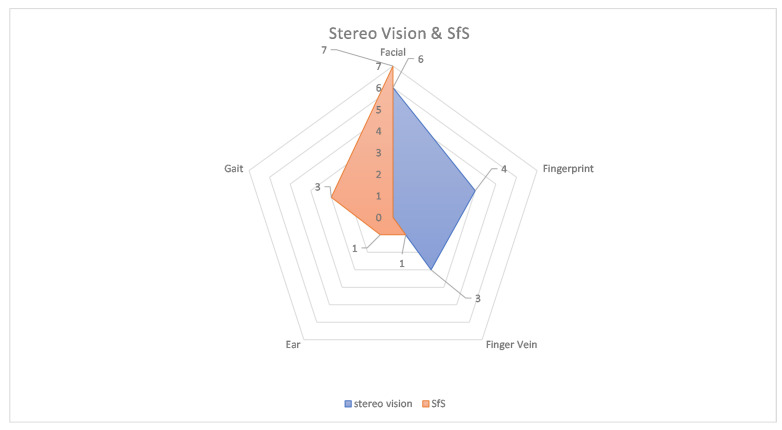
Applications with Stereo Vision and SfS by Biometric Category.

**Figure 12:**
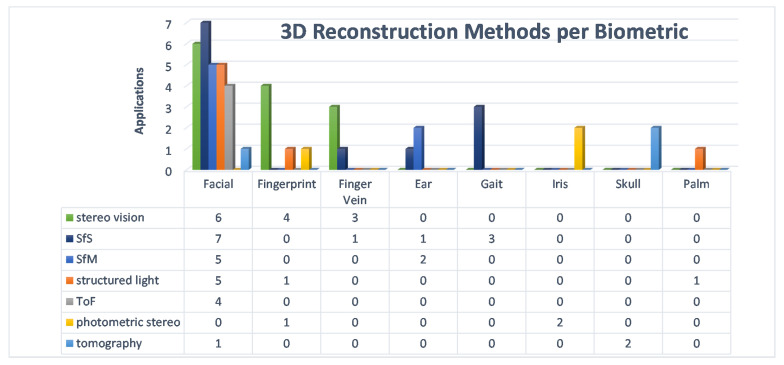
3D Reconstruction Methods per Biometric.

**Figure 13:**
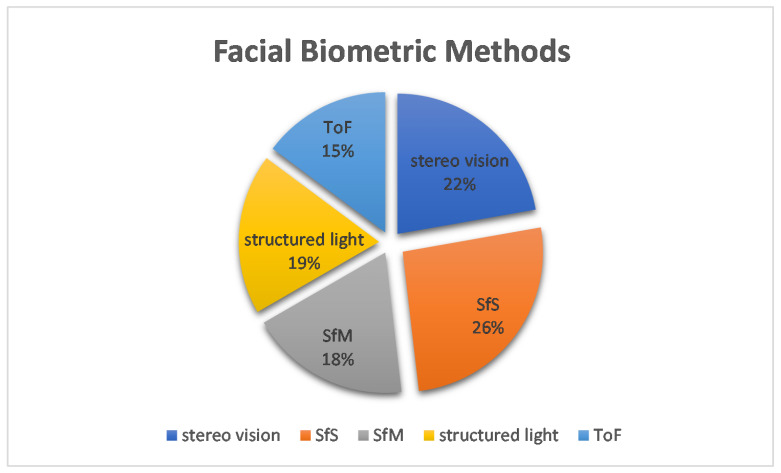
Facial Biometric Methods.

More interesting data can be obtained from further analysis of the methods. Figure 10 shows the percentages of each method in the number of applications. Here, it can be seen that stereo vision and SfS are the dominant methods with 27% and 25%, respectively. The combination of these two methods exceeds 50%. These two methods were used for facial, fingerprint, finger vein, ear and gait approaches. The exact number of applications is shown in Figure 11. The diagram shows that the facial uses both methods, as does the finger vein. In contrast, the gait and ear use SfS and fingerprints use stereo vision accordingly. To explore the relationship between the methods and each biometrics, a diagram is presented in Figure 12. Accordingly, gait, iris, skull and palm are associated with a method as follows: gait →SfS, iris →photometric stereo, skull →tomography, and palm →structured light. The most complicated of all is the facial, because the percentages are almost equal. To illustrate this, Figure 13 shows only the 3D facial biometric methods. According to the pie chart, although stereo vision and SfS predominate with a combined percentage of 48%, the other structured light, SfM, and ToF methods have similar numbers (19%, 18%, and 15%, respectively), and it is difficult to describe a pattern.

## 6. Discussion

This research has explored many aspects of 3D biometrics. First of all, facial recognition was by far the dominant 3D biometric. The percentage was 59.26% far enough from the others. New approaches are applied with different methods, from some traditional computer vision algorithms to state-of-the-art machine/deep learning methods. This particular biometric is almost saturated, and many experiments are being conducted. The percentages are already high, and the models are reliable and robust. On the other hand, there are also some weak points. The most recent one was exploited with COVID-19, where the face mask was an incomparable barrier. Any device with a built-in face recognition mechanism could not identify the correct person when the test subjects were wearing a mask. These types of reasons led to the need for alternative biometric methods. Such an example is the recent software upgrade of Apple’s Face ID, which is a built-in face recognition biometric mechanism. The user selects the option to activate the face recognition with a mask, and the software achieves it by focusing on different face parts such as the eyes and the area around them.

In addition, fusion techniques were proposed in some publications. More specifically, the performance was better when fusion was applied. Fusion can be applied in several ways. It can be applied between 2D and 3D images and has been shown to provide better results. In addition, it can be combined with different biometric features and opens a wide field for further research. There are a variety of combinations of biometric features that are practically limited to the capabilities of the capturing device that should capture the data simultaneously. Fusion can also be used for primary or supplemental use. The additional option can be activated when the results exceed a certain threshold. Such an addition to further research will provide a great framework for robust 3D biometrics.

In addition, the appropriate hardware plays an important role in achieving effective fusion. This literature analysis shows that some biometrics lack datasets, and to overcome this hurdle, researchers need to manufacture new experimental devices to capture the images. The choice of hardware depends on the choice of biometric. This causes additional effort if the experiment needs to combine several different biometrics. Another issue that needs to be pointed out is the lack of performance comparison between 2D and 3D biometrics under the same experimental conditions in the reviewed publications. Despite the potentially better performance of 3D biometrics models due to the larger number of features, they need to be compared to their 2D counterparts to confirm the need for the 3D transition.

To achieve this, future researchers should consider making their own hardware to allow for the simultaneous acquisition of 2D and 3D images. This will ensure that the images produced were created under the same conditions, allowing for a more reliable accuracy comparison. This multi-dimensional capability of the proposed device, in addition to the ability to compare, also provides the ability to merge 2D and 3D images. As mentioned earlier, this will lead to better results. There is another area that could benefit from this capability, which is the anti-spoofing.

The research area of anti-spoofing has a great impact on biometrics. This is another reason why researchers are moving biometrics to 3D, expanding its capabilities. According to the above analysis, some publications are choosing a 3D approach to defend against these types of attacks. Adding a 3D biometric module to a 2D device, or vice versa, offers the possibility of using that module as protection against spoofing. Further research could be conducted here to investigate how vulnerable each 3D biometric category is to spoofing and how each category can help protect against spoofing.

In summary, facial and fingerprints are the categories that benefit most from going 3D. Three-dimensional (3D) facial recognition is already being used on several devices for real-time 3D recognition. It has been proven that the additional features generated from depth information add robustness to the system. Its use in everyday devices, such as smartphones, shows that hardware development is on the cutting edge to support these technologies. On the other hand, despite the fact that there are many approaches to 3D fingerprinting and the additional data have led to great results, the 3D fingerprint system is still far from being used commercially. There are limitations in the capture devices, as fingerprints are already captured with small devices. However, the development of contactless methods is likely to solve this problem. Although it is not obvious that going 3D will automatically give better results than 2D approaches, advanced 3D reconstruction algorithms and hardware should be used to achieve great performance. Therefore, it is worth investing in new algorithms and improving hardware so that the other biometric (other than facial and fingerprint) also provide reliable recognition systems.

Finally, as mentioned earlier, there are datasets for some 3D biometrics and not for others, as there are some ethical issues regarding the release of these data. These data are very sensitive, and in some countries, their publication is prohibited by local laws. Some research teams have developed a dataset but have not shared it with the scientific community. This is an obstacle for prospective research, which needs to create the dataset to conduct the desired experiments. To overcome this hurdle, more efforts should be made to publish the appropriate datasets. Finally, there has not been enough research effort to generalize the 3D reconstruction algorithms, and more research should be conducted to share the state-of-the-art algorithms among different 3D biometrics.

## 7. Conclusions

This paper is a literature review of 53 papers that attempt to present the various aspects of the transition to 3D biometrics. The papers were selected based on strict criteria, which are described in Section 3. The search targeted 3D biometrics under 3D reconstruction since 2011.

After a comprehensive review of the selected publications, the initial statistical results showed that facial recognition was the most commonly used 3D biometric in this particular research area. The other seven categories were fingerprint, finger vein, ear, iris, gait, skull and palm. These can be classified into three main categories: face, hand and gait. Related work was also presented in Section 2, showing that some of the biometrics have not yet been reviewed, and further research needs to be conducted.

Then, in Section 5, the results of the conducted analysis are presented. The available 3D biometrics databases and the state of the art per category are presented in Table 2 and Table 3, respectively. Summarizing the results, it becomes clear in which area more research should be conducted. This is the creation of multimodal biometrics that combine 2D with 3D and mixing the different categories such as the Tharewal et al. multimodal approach with face and ear [148]. In addition, further research should be conducted on how resistant each category is to spoofing and how they offer new methods to deal with it. Finally, further research should be conducted on how the 3D reconstruction algorithms can be generalized to 3D biometrics.

As for future work, a framework should be described in which each new 3D biometric research should include metrics about the corresponding 2D images. To this end, devices should include 2D capabilities. In addition, more datasets for less common biometric features should be created to make it easier for future researchers to conduct experiments. These points are crucial and will be the key factor for further improvements so that the necessity for going 3D can be proven.

## Figures and Tables

**Table 1:** Literature Table.

Biometric	Major Category	Percentage (%)	References
Facial	Face	59.26	[42,43,44,45,46,47,48,49,50,51,52,53,54,55,56,57,58,59,60,61,62,63,64,65,66,67,68,69,70,71,58,71]
Fingerprint	Hand	11.11	[72,73,74,75,76,77]
Finger Vein	Hand	7.41	[78,79,80,81]
Ear	Face	5.56	[45,82,83]
Iris	Face	5.56	[84,85,86]
Gait	Gait	5.56	[87,88,89]
Skull	Face	3.70	[43,90]
Palm	Hand	1.85	[91]

**Table 2:** Three-Dimensional (3D) Biometrics Dataset Table.

Dataset	Biometric	Number of Images	Classes	Year	Reference
AFLW	Facial	21,997	25,993	2011	[123]
3D-MAD	Facial	76,500	17	2013	[124]
Bosphorus	Facial	4666	105	2008	[125]
BU-3DFE	Facial	2500	100	2006	[126]
BU-4DFE	Facial	60,600	101	2013	[127]
Feret	Facial	14,126	1199	2000	[128]
FRGC	Facial	50,000	12,500	2004	[129]
Morpho	Facial	200	20	2013	[130]
The Photoface Database	Facial	7356	261	2011	[131]
LFW	Facial	13,233	5749	2019	[132]
Youtube Faces	Facial	3245 (videos)	1595	2011	[133]
Pie	Facial	75,000	337	2002	[134]
UHDB11	Facial	1656	23	2013	[135]
IIT-Kanpur	Ear	465	125	2012	[136]
AMI	Ear	700	100	2008	[137]
UCR	Ear	902	155	2007	[138]
UND	Ear	1686	415	2007	[139]
XM2VTS	Ear	1180 (videos)	295	2013	[140]
AVAMVG	Gait	200 (videos)	20	2014	[141]
KY4D	Gait	168 (videos)	42	2014	[142]
i3DPost	Gait	768 (videos)	8	2009	[143]
MuHAVi	Gait	136 (videos)	14	2010	[144]
IXMAS	Gait	550 (videos)	10	2006	[145]
SCUT LFMB-3DPVFV	Finger Vein	16,848	702	2022	[81]
IIT Iris Database	Iris	1120	224	2007	[146]
Hong Kong Polytechnic 3D	Fingerprint	1560	260	2016	[147]

**Table 3:** Three-Dimensional (3D) Biometrics State of the Art.

Title	Biometric	Score	Dataset	Year	Reference
Verifying kinship from rgb-d face data	Facial	95% (accuracy)	Kin3D	2020	[59]
A novel 3D ear reconstruction method using a single image	Ear	manual	UND	2012	[98]
A 3D iris scanner from a single image using convolutional neural networks	Iris	99.8% (accuracy)	98,520 iris	2020	[85]
An accuracy assessment of forensic computerized facial reconstruction employing cone-beam computed tomography from live subjects	Skull	0.31 mm (error)	3 humans	2012	[43]
3D fingerprint recognition based on ridge–valley guided 3D reconstruction and 3D topology polymer feature extraction	Fingerprint	98% (accuracy)	DB1, DB2	2019	[77]
Endowing rotation invariancefor 3D finger shape and vein verification	Finger Vein	2.61 (ER%)	3DPVFV	2022	[81]
Biometric recognition of people by 3D hand geometry	Palm	0.0018 (RMSE)	3 palms	2013	[91]
Model-based interpolation for continuous human silhouette images by height-constraint assumption	Gait	95%	KY 4D	2020	[89]

## Data Availability

Not applicable, the study does not report any data.
